# A Novel Recombinant Influenza Virus Neuraminidase Vaccine Candidate Stabilized by a Measles Virus Phosphoprotein Tetramerization Domain Provides Robust Protection from Virus Challenge in the Mouse Model

**DOI:** 10.1128/mBio.02241-21

**Published:** 2021-11-23

**Authors:** Shirin Strohmeier, Fatima Amanat, Xueyong Zhu, Meagan McMahon, Meagan E. Deming, Marcela F. Pasetti, Kathleen M. Neuzil, Ian A. Wilson, Florian Krammer

**Affiliations:** a Department of Microbiology, Icahn School of Medicine at Mount Sinai, New York, New York, USA; b Department of Biotechnology, University of Natural Resources and Life Sciences, Vienna, Austria; c Graduate School of Biomedical Sciences, Icahn School of Medicine at Mount Sinai, New York, New York, USA; d Department of Integrative Structural and Computational Biology, The Scripps Research Institute, La Jolla, California, USA; e Skaggs Institute for Chemical Biology, The Scripps Research Institute, La Jolla, California, USA; f Center for Vaccine Development and Global Health, University of Maryland School of Medicine, Baltimore, Maryland, USA; g Department of Pathology, Molecular and Cell Based Medicine, Icahn School of Medicine at Mount Sinai, New York, New York, USA; St. Jude Children's Research Hospital

**Keywords:** influenza vaccine, neuraminidase, vaccine design

## Abstract

Current seasonal influenza virus vaccines do not induce robust immune responses to neuraminidase. Several factors, including immunodominance of hemagglutinin over neuraminidase, instability of neuraminidase in vaccine formulations, and variable, nonstandardized amounts of neuraminidase in the vaccines, may contribute to this effect. However, vaccines that induce strong antineuraminidase immune responses would be beneficial, as they are highly protective. Furthermore, antigenic drift is slower for neuraminidase than for hemagglutinin, potentially providing broader coverage. Here, we designed stabilized recombinant versions of neuraminidase by replacing the N-terminal cytoplasmic domain, transmembrane, and extracellular stalk with tetramerization domains from the measles or Sendai virus phosphoprotein or from an Arabidopsis thaliana transcription factor. The measles virus tetramerization domain-based construct, termed N1-MPP, was chosen for further evaluation, as it retained antigenicity, neuraminidase activity, and structural integrity and provided robust protection *in vivo* against lethal virus challenge in the mouse model. We tested N1-MPP as a standalone vaccine, admixed with seasonal influenza virus vaccines, or given with seasonal influenza virus vaccines but in the other leg of the mouse. Admixture with different formulations of seasonal vaccines led to a weak neuraminidase response, suggesting a dominant effect of hemagglutinin over neuraminidase when administered in the same formulation. However, administration of neuraminidase alone or with seasonal vaccine administered in the alternate leg of the mouse induced robust antibody responses. Thus, this recombinant neuraminidase construct is a promising vaccine antigen that may enhance and broaden protection against seasonal influenza viruses.

## INTRODUCTION

Influenza A viruses belong to the family *Orthomyxoviridae* and are enveloped, negative-sense, single-stranded RNA viruses. Their genome consists of 8 segments, which encode at least 11 structural and nonstructural proteins (1). The main antigenic targets for humoral immunity are the two major glycoproteins on the viral surface, the hemagglutinin (HA) and the neuraminidase (NA) (2). The immunodominant HA appears to be more susceptible to mutation and undergoes frequent antigenic drift, particularly in its strongly immunodominant head domain. In contrast, the immunosubdominant NA is less divergent and subject to less antigenic drift over time (3–6). The NA is a homotetramer and is classified as a type 2 membrane protein (7). Each monomer consists of a cytoplasmic tail, a transmembrane domain, a hypervariable stalk domain, and a globular head domain. The globular head domain of each monomer contains an enzymatic active site, whose activity plays an integral role during the viral replication cycle. In particular, the NA cleaves sialic acid residues from newly formed virions as they bud from the viral host cell surface (7, 8). Furthermore, NA destroys decoy receptors in the mucosal fluids, facilitating viral engagement of target cells (9, 10). The NA is therefore the main target of antiviral drugs for the treatment of influenza virus infections (e.g., oseltamivir, zanamivir, peramivir, etc.). The drugs can improve influenza symptoms when administered at early stages of infection through preventing detachment of progeny virions from the host cell surface, leading to an accumulation of virus aggregates and inhibition of further spread (8). Despite the availability of antiviral therapeutics, influenza virus infections are still a major public health concern, burden the health system through rising hospitalizations, and lead to 290,000 to 650,000 deaths worldwide each year (11). Seasonal vaccines are the major preventive defense against influenza viruses and are administered on an annual basis, inducing a narrow, HA-targeted, and mostly strain-specific immune response (2). Influenza virus vaccines are usually standardized to the amount of HA that they contain and are formulated either as a trivalent inactivated influenza vaccine (TIV), containing H1N1, H3N2, and either influenza B/Yamagata/16/88-like or B/Victoria/2/87-like lineage strains, or as a quadrivalent inactivated influenza vaccine (QIV), containing H1N1, H3N2, B/Yamagata/16/88-like, and B/Victoria/2/87-like lineage strains. However, if the vaccine viruses are mismatched to circulating viruses due to antigenic drift or egg adaptation of vaccine strains, the vaccine effectiveness is significantly reduced (12–17).

Recently, the NA has reemerged as an attractive antigenic target for vaccine development since it harbors broadly conserved epitopes and is less affected by antigenic drift (3). Studies in animal models and humans show that antibodies against the NA correlate with protection and reduced viral shedding (18–31). Unfortunately, the NA content of current vaccines is not standardized, and its structural integrity in vaccine formulations is likely suboptimal. In addition, it has been suggested that the NA becomes immunosubdominant in the presence of HA (32–35). In fact, the anti-NA antibody response after vaccination with inactivated vaccines or live attenuated vaccines is mediocre at best (18, 36). It has been suggested that stable, recombinant NA could be added to the current seasonal vaccines or that NA could be administered on its own as a means of addressing the suboptimal anti-NA responses elicited by traditional vaccination (33–35, 37, 38). Purification of NA from virions is difficult, but recombinant full-length NA has been expressed and purified from insect cells. While this procedure was successful, it had a low yield and the stability of the resulting NA preparations was unclear (39). In contrast, expression of the NA head domain, which harbors the majority, if not all, of the NA protective epitopes, as soluble protein produces high yields. NA tetramerization is usually mediated by the transmembrane domain, disulfide bond formation in the variable stalk domain, and interprotomer interactions in the head domain (40, 41). Importantly, multimerization of NA has been shown to be required for induction of protective immune responses (25, 42).

However, it is a challenge to produce a stable, tetrameric, enzymatically active, recombinant NA protein. Previously, a human-derived vasodilator-stimulated phosphoprotein (VASP) tetramerization domain was used to produce stable recombinant NA, and these constructs showed full protection *in vivo* and high enzymatic activity and stability *in vitro* (18, 43). However, the use of a human protein based sequence is problematic for vaccine development, since concerns regarding autoimmune reactions could arise. Here, we generated stable soluble recombinant NA constructs of the N1 subtype (A/Michigan/45/15 H1N1 [Mich15] strain) using the baculovirus expression system. The constructs were engineered to contain either a measles virus phosphoprotein tetramerization domain (N1-MPP), a Sendai virus phosphoprotein tetramerization domain (N1-SPP), or a leucine zipper from the SEPPALLATA-like MADS domain transcription factor derived from Arabidopsis thaliana (N1-SEP). All were fused to the globular head domain of the NA, with some resulting in a fully functional and stable tetrameric NA glycoprotein. By using this approach, we demonstrate that the N1-MPP/N1-SPP, but not the N1-SEP, constructs are potential vaccine candidates that might lead to enhanced antibody responses toward the NA. Vaccines that incorporate standardized amounts of NA might lead to broader breadth and increased protection when administered as a standalone vaccine or as a supplement to current seasonal TIVs/QIVs.

## RESULTS

### N1-MPP and N1-SPP form stable tetramers and exhibit full enzymatic activity.

**FIG 1:**
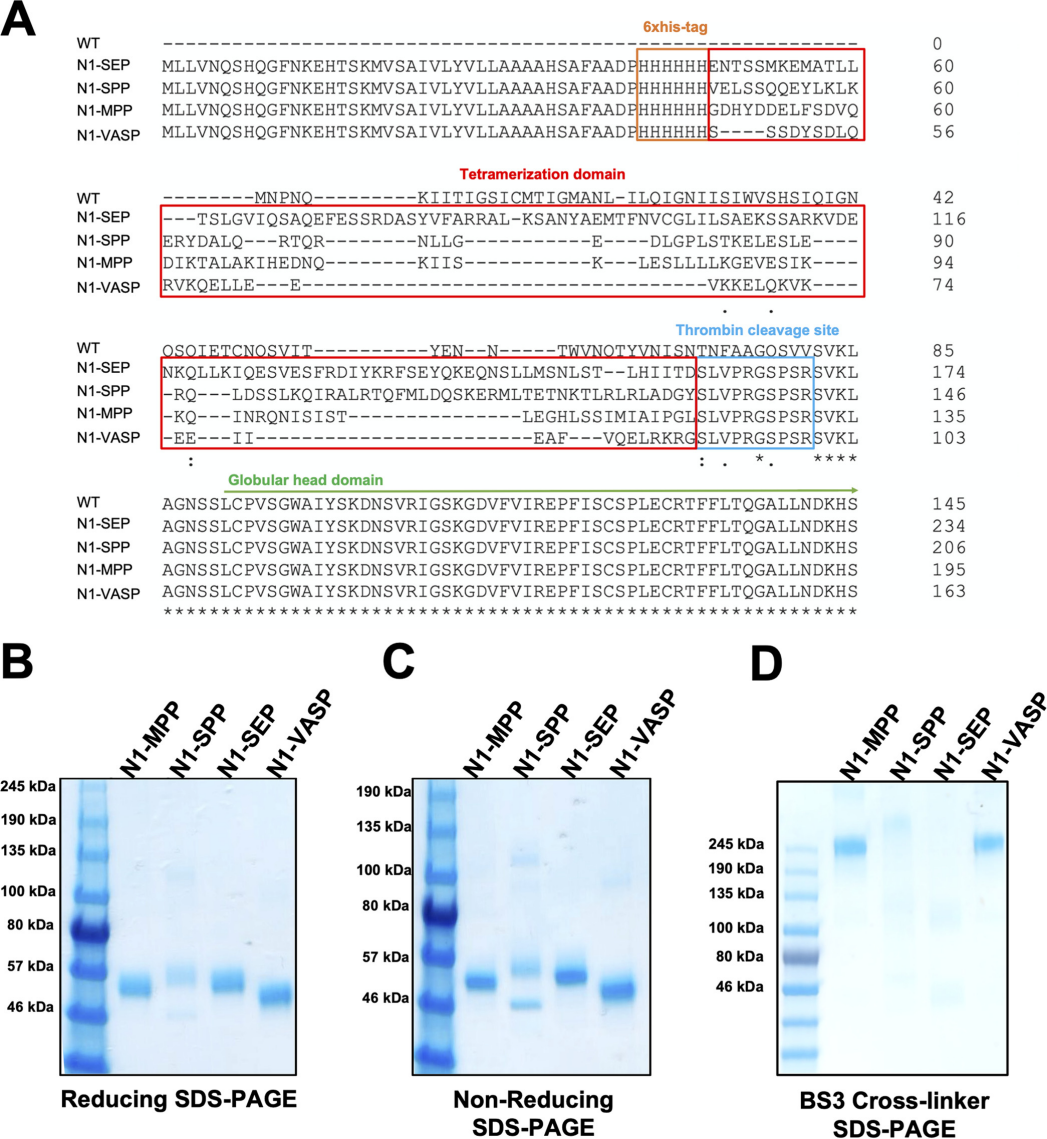
Sequences and structural analysis of N1-MPP, N1-SPP, and N1-SEP. (A) Sequence alignment between N1-SEP, N1-SPP, N1-MPP, and N1-VASP. Highlighted are the hexahistidine purification tag, the respective tetramerization domain, the thrombin cleavage site, and the start of the globular head domain. Asterisks indicate identical amino acids, colons indicate strongly similar amino acids, and dots indicate weak similarity. (B) SDS-PAGE under reducing conditions. N1-MPP, N1-SEP, and the positive control (N1-VASP) show monomeric structures at 55 kDa. N1-SPP shows an additional band at 46 kDa. (C) SDS-PAGE under nonreducing conditions. N1-MPP, N1-SEP, and N1-VASP (positive control) show monomeric structures at 55 kDa. N1-SPP shows additional bands at 46 kDa and 115 kDa. (D) SDS-PAGE using a BS3 cross-linker. N1-MPP, N1-SPP, and N1-VASP (positive control) show tetrameric structures at 245 kDa, whereas N1-SEP appears to be unable to tetramerize.

To generate constructs that allow for high, stable expression levels of recombinant tetrameric neuraminidase, we cloned sequences encoding the measles or Sendai virus phosphoprotein tetramerization domains or a leucine zipper found in an Arabidopsis thaliana transcription factor into the 5′ end of the sequence coding for the N1 NA head domain of A/Michigan/45/15 to generate fusion proteins. The constructs were then expressed in insect cells and purified via an N-terminal hexahistidine tag (Fig. 1A).

First, the structural integrity of the recombinant proteins was verified by visualizing them by sodium dodecyl-sulfate polyacrylamide gel electrophoresis (SDS-PAGE). Under reducing conditions, we observed that N1-MPP and N1-SEP showed a clear band at around 55 kDa, which is the expected size of an N1 monomer head plus the tetramerization domain. However, N1-SPP had an additional band at 46 kDa, indicating degradation/cleavage of the protein (Fig. 1B). In the absence of reducing reagents, N1-SPP displayed an additional band at around 115 kDa, suggesting the formation of multimeric, potentially dimeric, structures (Fig. 1C), whereas for N1-MPP and N1-SEP, only monomers were visible. By adding a BS3 cross-linker, which cross-links primary amines, N1-MPP and N1-SPP, as well as the positive-control N1-VASP, formed tetramers at around 245 kDa, whereas N1-SEP appears unable to tetramerize and displayed two bands at around 100 and 50 kDa (Fig. 1D). Since N1-SEP was unable to form a stable tetramer, a key requirement for immunogenicity, it was excluded from future experiments.

**FIG 2:**
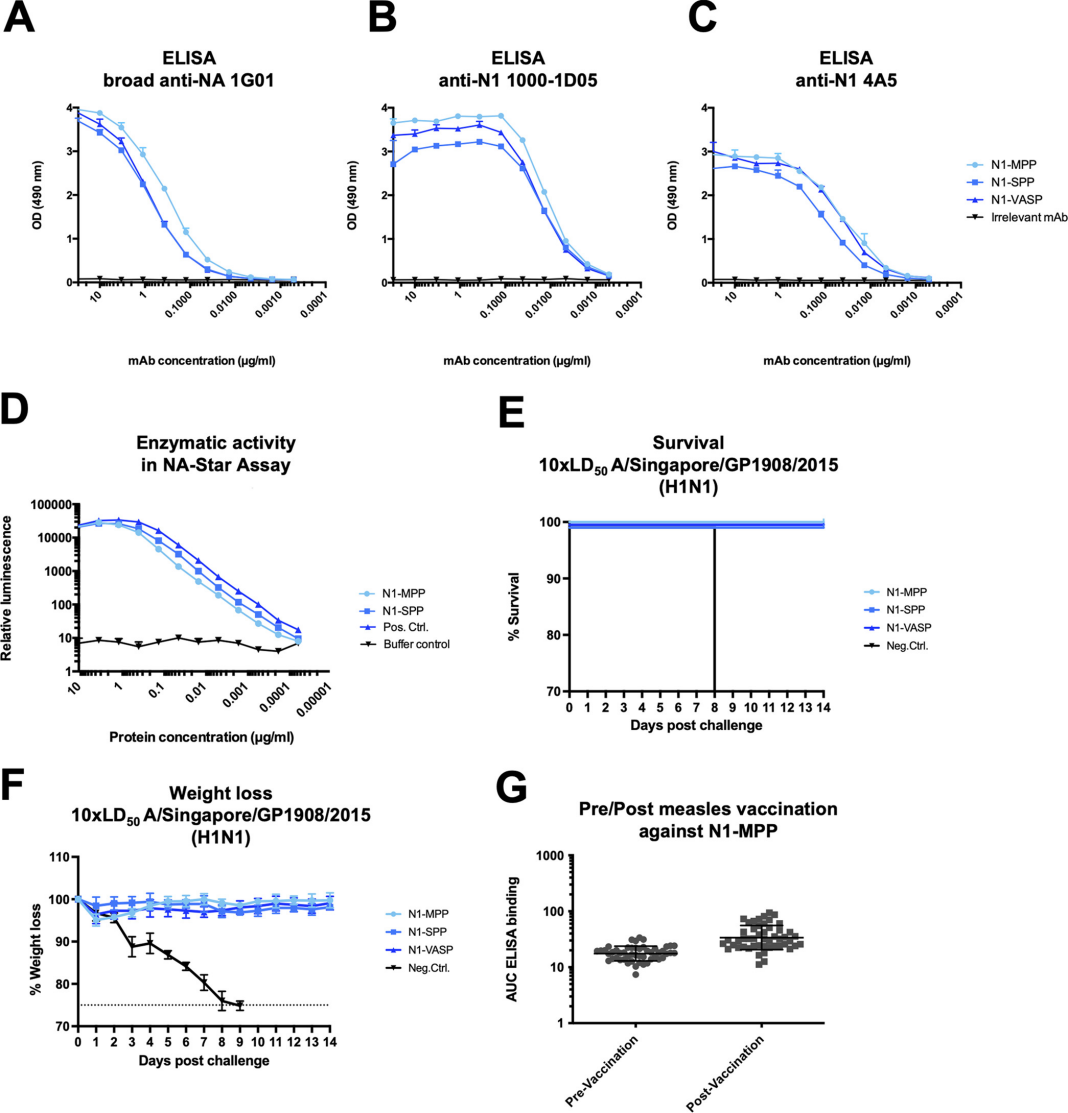
Antigenicity and enzymatic activity of N1-MPP and N1-SPP. (A) ELISA against recombinant N1-MPP, N1-SPP, N1-VASP, and an irrelevant protein (B-HA) using the human broad anti-NA antibody 1G01. (B) ELISA against recombinant N1-MPP, N1-SPP, N1-VASP, and an irrelevant protein (B-HA) using the human anti-N1 antibody 1000-1D05. (C) ELISA against recombinant N1-MPP, N1-SPP, N1-VASP, and an irrelevant protein (B-HA) using the mouse anti-N1 antibody 4A5. (D) Enzymatic activity of N1-MPP, N1-SPP, and N1-VASP determined via the NA-Star assay. (E and F) Weight loss kinetics (E) and survival (F) of a pilot vaccination study with N1-MPP, N1-SPP, N1-VASP, and an irrelevant protein (B-Malaysia HA) after challenge with 10× the LD_50_ of A/Singapore/GP1908/2015 (H1N1). (G) Cross-reactivity testing against the measles virus tetramerization domain. Pre- and postmeasles serum obtained from children in Burkina Faso was tested against recombinant N1-MPP.

To verify the correct display of antigenic epitopes, an enzyme-linked immunosorbent assay (ELISA) was conducted using monoclonal antibodies (MAbs) 1G01 (Fig. 2A), 1000-1D05 (Fig. 2B), and 4A5 (Fig. 2C). Both 1G01 and 1000-1D05 are human MAbs that are protective *in vivo*, and therefore retention of their binding seemed to be important (18, 44, 45). MAb binding to N1-MPP and N1-SPP demonstrated a binding pattern similar to that of N1-VASP, suggesting that the probed epitopes are presented in a native-like conformation. An anti-Lassa antibody, KL-AV-1A12, was used as a negative control. To assess the enzymatic activities of the proteins, an NA-Star assay was performed. N1-MPP and N1-SPP exhibited high enzymatic activity, comparable to that of the positive control, N1-VASP (Fig. 2D), which indicates correct folding of the proteins. In a pilot vaccination study, both antigens were tested *in vivo*, and weight loss (Fig. 2E) and survival (Fig. 2F) after a 10× 50% lethal dose (LD_50_) challenge with A/Singapore/GP1908/2015 (H1N1) were monitored over 14 days. N1-VASP was included as a positive control, and an irrelevant protein (B-HA from B/Malaysia/2506/2004) was included as a negative control. In all three groups, N1-MPP, N1-SPP, and N1-VASP, no significant weight loss was detectable.

While both N1-SPP and N1-MPP were highly protective in the mouse model, we chose to move N1-MPP forward in development, since it showed no degradation/cleavage in the initial characterization. However, since this domain is derived from measles virus and a high percentage of the human population is vaccinated against measles, we wanted to determine the degree of response to this domain triggered by measles vaccination. For this, sera obtained from children pre- and post-measles vaccination were tested against the recombinant N1-MPP protein via ELISA. This analysis revealed very low absolute serum antibody reactivity to the construct (close to the limit of detection) and negligible induction of antibodies after measles vaccination (Fig. 2G).

### N1 NA expressed as N1-MPP resembles previously solved N1 NA structures.

**FIG 3:**
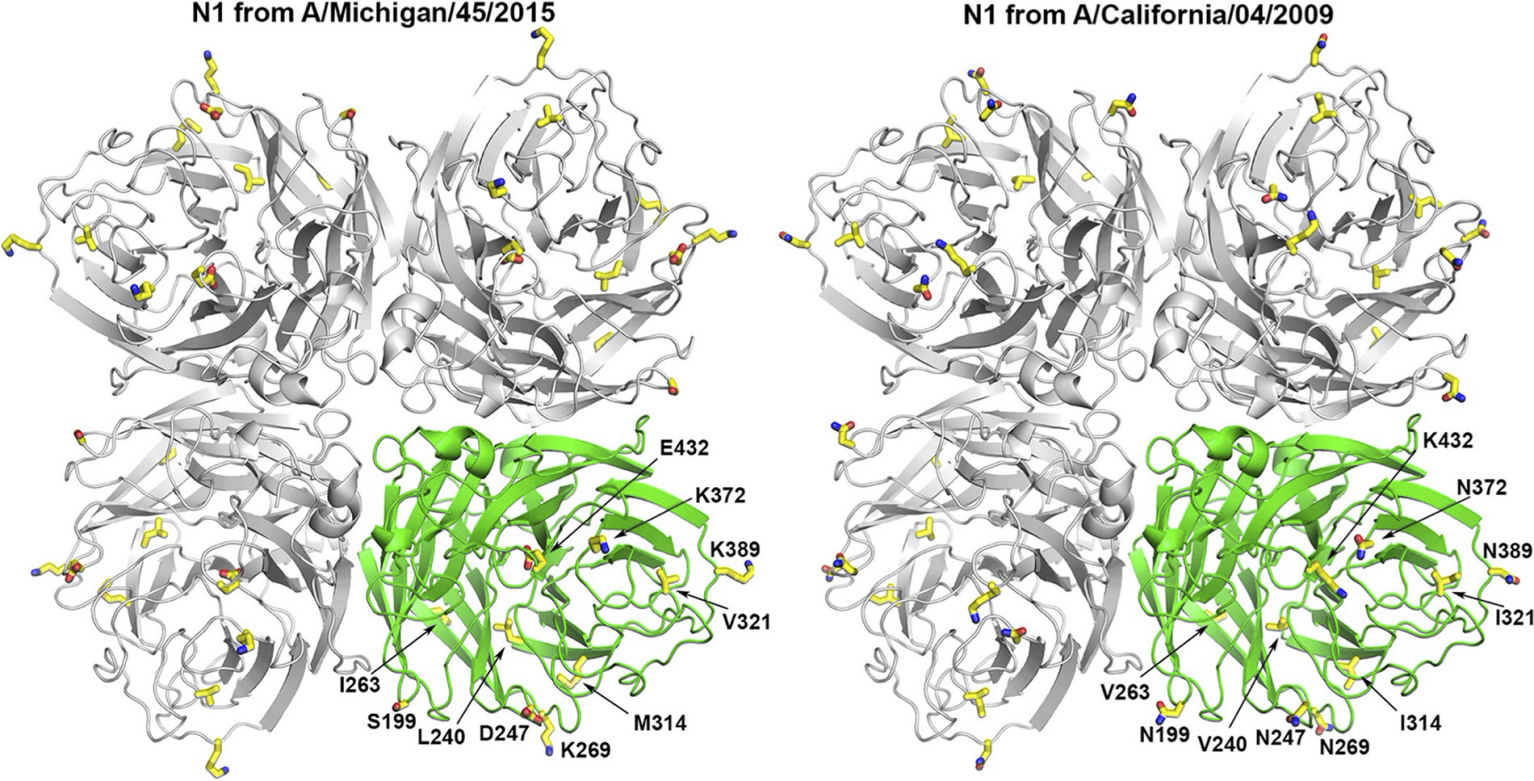
Crystal structures of the N1 NA head from recombinant N1-MPP and the N1 head structure from A/California/04/2009 (H1N1) (PDB accession no. 3NSS). Each N1 tetramer is colored in gray, with one protomer colored in green. The 10 residues that are not conserved between these two N1 NAs in their head (aa 82 to 471) are shown in sticks and colored with yellow carbon, red oxygen, and blue nitrogen atoms.

To verify the correct conformation of the N1 NA head domain (residues 82 to 471, N2 numbering) from A/Michigan/45/2015 (H1N1) in the recombinant N1-MPP, the N1-MPP was first expressed in Sf9 insect cells and purified, and the N1 head was obtained by removing the MPP tetramerization domain from N1-MPP with thrombin. The crystal structure of the N1 NA head was determined to a 2.90-Å resolution (see Table S1 in the supplemental material). The overall structure shows a typical box-shaped tetramer and resembles other published N1 structures, such as the N1 NA head from A/California/04/2009 (H1N1) (46), with a root mean square deviation (RMSD) value of 0.28 Å for all C_α_ atoms (Fig. 3). Ten residues that are different between A/Michigan/45/2015 and A/California/04/2009 (H1N1) are located over the outer region of the head domain, where residues 199, 247, 263, 269, 314, 372, 389, and 432 are solvent exposed and residues 240 and 321 are buried but have similar hydrophobic residues (Fig. 3).

10.1128/mBio.02241-21.1 TABLE S1 Data collection and refinement statistics. Download Table S1, DOCX file, 0.01 MB. Copyright © 2021 Strohmeier et al. 2021 Strohmeier et al. https://creativecommons.org/licenses/by/4.0/ This content is distributed under the terms of the Creative Commons Attribution 4.0 International license.

### Vaccination with recombinant N1-MPP provides full protection against influenza virus challenge *in vivo*, but admixing with QIV reduces anti-NA responses.

**FIG 4:**
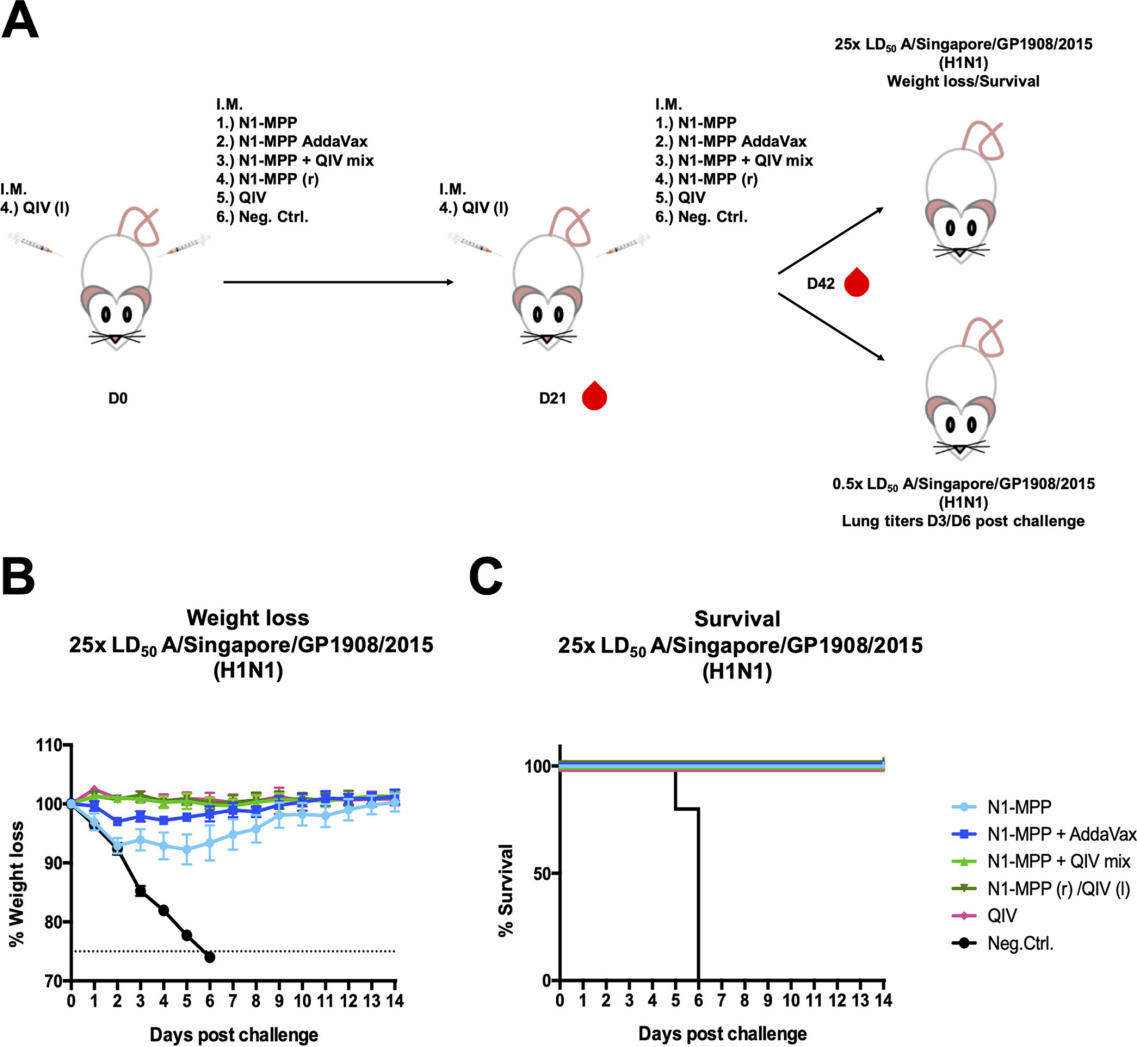
Testing the protective potential of N1-MPP *in vivo*. (A) Vaccination scheme for testing the protective potential of N1-MPP *in vivo* as a standalone vaccine or as a supplement for the seasonal QIV. Female 6- to 8-week-old BALB/c mice were vaccinated in a prime/boost regimen and then challenged either with 25× the LD_50_ of A/Singapore/GP1908/2015 (H1N1) so that we could observe weight loss and survival or with 0.5× the LD_50_ of A/Singapore/GP1908/2015 (H1N1) so that we could determine viral titers in lung tissue at day 3 and day 6 postchallenge. Additionally, mice were bled on day 21 (D21) and day 42 for serological analysis. To assess the protective potential of N1-MPP, the protein was administered either as a standalone vaccine or in combination with a seasonal QIV (Flucelvax). I.M., intramuscular vaccination. (B) Weight loss curve after challenge with 25× the LD_50_ of A/Singapore/GP1908/2015 (H1N1). N1-MPP mice experienced the most weight loss, i.e., around 10%. The dotted line indicates 75% of the initial weight, the endpoint for weight loss. (C) Survival curve. All groups were protected against mortality. The negative-control mice succumbed to infection by day 6.

Since current vaccine formulations are standardized based on the HA content, the antibody response induced by seasonal TIV/QIV toward the NA is usually suboptimal (18, 36). To test if recombinant NA protein could induce and broaden a protective immune response, we vaccinated mice (5 per group) in a prime/boost regimen; N1-MPP was administered either as a standalone vaccine (adjuvanted and nonadjuvanted) or as a supplement to seasonal QIV (Flucelvax, matched to the challenge strain) or together with seasonal QIV but in the opposite leg of the mouse (Table 1; Fig. 4A). Following vaccination, mice were challenged with QIV-matched influenza A/Singapore/GP1908/2015 (H1N1) virus at 25× the LD_50_. While vaccination with the N1-MPP construct itself induced a fully protective immune response, mice experienced a 10% weight loss. By adjuvanting the N1-MPP with AddaVax, mice were fully protected against mortality and morbidity. The same was true when the QIV (matched to the challenge strain) was supplemented with N1-MPP or when the QIV and N1-MPP were given at the same time, but in different legs of the mouse. As a negative control, mice were vaccinated with irrelevant influenza B virus HA protein (Fig. 4B and C), and all of these mice succumbed to infection on days 5 and 6. This experiment was repeated with the same outcome (Fig. S1).

**TABLE 1:** Vaccination regimen for N1-MPP as a standalone vaccine or as a supplement to the seasonal QIV^*a*^

Group	Prime	Boost
1	3 μg N1-MPP	3 μg N1-MPP
2	3 μg N1-MPP + AddaVax	3 μg N1-MPP + AddaVax
3	3 μg N1-MPP + QIV mix	3 μg N1-MPP + QIV mix
4	3 μg N1-MPP in the right leg/QIV in the left leg	3 μg N1-MPP in the right leg/QIV in the left leg
5	QIV	QIV
6	3 μg irrelevant HA protein	3 μg irrelevant HA protein

a The challenge was 25× the LD_50_ of A/Singapore/GP1908/2015 (H1N1).

**FIG 5:**
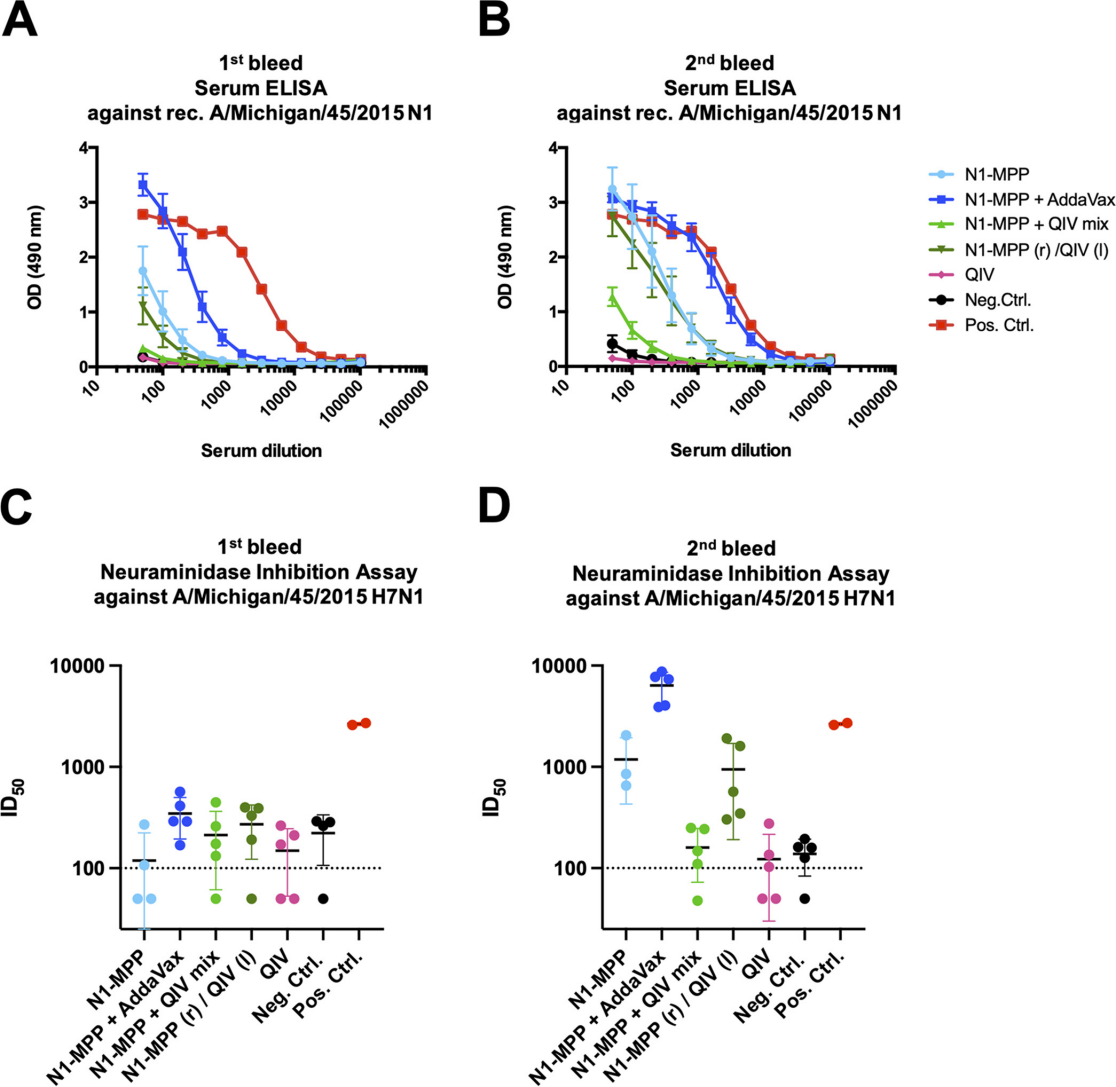
Serological assessment of serum samples obtained after N1-MPP vaccination. Serum samples were collected before the boost and before the challenge. (A) ELISA against recombinant (rec.) N1-VASP protein with serum obtained before the boost. N1-MPP plus AddaVax showed the highest antibody response. (B) ELISA against recombinant N1-VASP protein with serum obtained before the challenge. N1-MPP plus AddaVax, N1-MPP administered in the right leg with the QIV administered in the left leg, and N1-MPP showed high binding against N1. An anti-His antibody was used as a positive control. (C) NI assay using a reassortant H7N1 virus containing the N1 of A/Michigan/45/2015. Serum obtained before the boost was used. No statistical significance between the groups was found (the positive control was excluded from analysis since it is only a technical assay control). (D) NI assay using serum collected before the challenge. In a one-way ANOVA corrected for multiple comparisons, only the N1-MPP plus AddaVax group was statistically different from all other groups (*P* < 0.0001; the positive control was excluded from analysis, since it is only a technical assay control). The human antibody 1G01 was used as a positive control in panels C and D, and the dotted line represents the limit of detection. Negative samples were set to half of the limit of detection for graphing and analysis purposes. (A to D) Samples in A and B as well as in C and D were run at the same time, and the positive control was shared.

10.1128/mBio.02241-21.2 FIG S1 Repeat of *in vivo* vaccination study to assess the protective potential of N1-MPP. The experiments which can be seen in Fig. 4 and 5 have been conducted twice. (A) Weight loss curve after challenge with 25× the LD_50_ of A/Singapore/GP1908/2015 (H1N1). N1-MPP-vaccinated mice experienced the most weight loss (around 10%). (B) Survival curve. All groups were protected against mortality. The negative-control mice succumbed to infection by day 6. (C) ELISA against recombinant N1-VASP protein with serum obtained before challenge. N1-MPP plus AddaVax, N1-MPP (r)/QIV (l), and N1-MPP showed high binding against N1-VASP. (D) NI assay using serum collected before challenge. The N1-MPP plus AddaVax and N1-MPP (r)/QIV (l) groups reached 100% NI. Download FIG S1, TIF file, 0.4 MB. Copyright © 2021 Strohmeier et al. 2021 Strohmeier et al. https://creativecommons.org/licenses/by/4.0/ This content is distributed under the terms of the Creative Commons Attribution 4.0 International license.

### Serum antibodies induced after N1-MPP vaccination exhibit strong binding and NI activity.

To assess the characteristics of serum antibodies obtained after N1-MPP vaccination, the capacity to bind to recombinant N1-VASP protein was tested. N1-VASP was used as the substrate to prevent the detection of antibodies that bind to the MPP tetramerization domain. Serum was obtained 3 weeks postpriming as well as prior to challenge. After the prime vaccination, a strong antibody response was detectable only in the group that received N1-MPP adjuvanted with AddaVax, with weaker responses to N1-MPP and to N1-MPP (in the right leg [r]) and the QIV (in the left leg [l]) (Fig. 5A). After the second vaccination, the antibody reactivities of the N1-MPP and N1-MPP (r)/QIV (l) groups increased substantially (Fig. 5B). The same trend was seen in terms of neuraminidase inhibition (NI) titers when an H7N1 virus, containing an exotic H7 HA and the N1 NA of A/Michigan/45/2015, was used. Vaccination with N1-MPP AddaVax induced a moderate level of NA inhibition after one vaccination (Fig. 5C). After the second dose, N1-MPP AddaVax NA inhibition increased substantially, and the N1-MPP and N1-MPP (r)/QIV (l) groups now exhibited moderate NA inhibition (Fig. 5D). Interestingly, when the N1-MPP was admixed with the QIV, the immune response was much lower than giving N1-MPP and QIV in different legs, reminiscent of earlier reports by Johansson et al. (32) and Johansson and Kilbourne (34) regarding immunodominance of the HA. Interestingly, this effect was asymmetric, and the response against H1 HA was not impacted (Fig. S2).

10.1128/mBio.02241-21.3 FIG S2 ELISA against the recombinant H1 (A/Michigan/45/2015) protein with sera of mice vaccinated with N1-MPP. The antibody response to the HA component within the QIV is not suppressed by admixing N1-MPP. Download FIG S2, TIF file, 0.1 MB. Copyright © 2021 Strohmeier et al. 2021 Strohmeier et al. https://creativecommons.org/licenses/by/4.0/ This content is distributed under the terms of the Creative Commons Attribution 4.0 International license.

### Vaccination with N1-MPP leads to a reduction of viral load in lung tissues.

**FIG 6:**
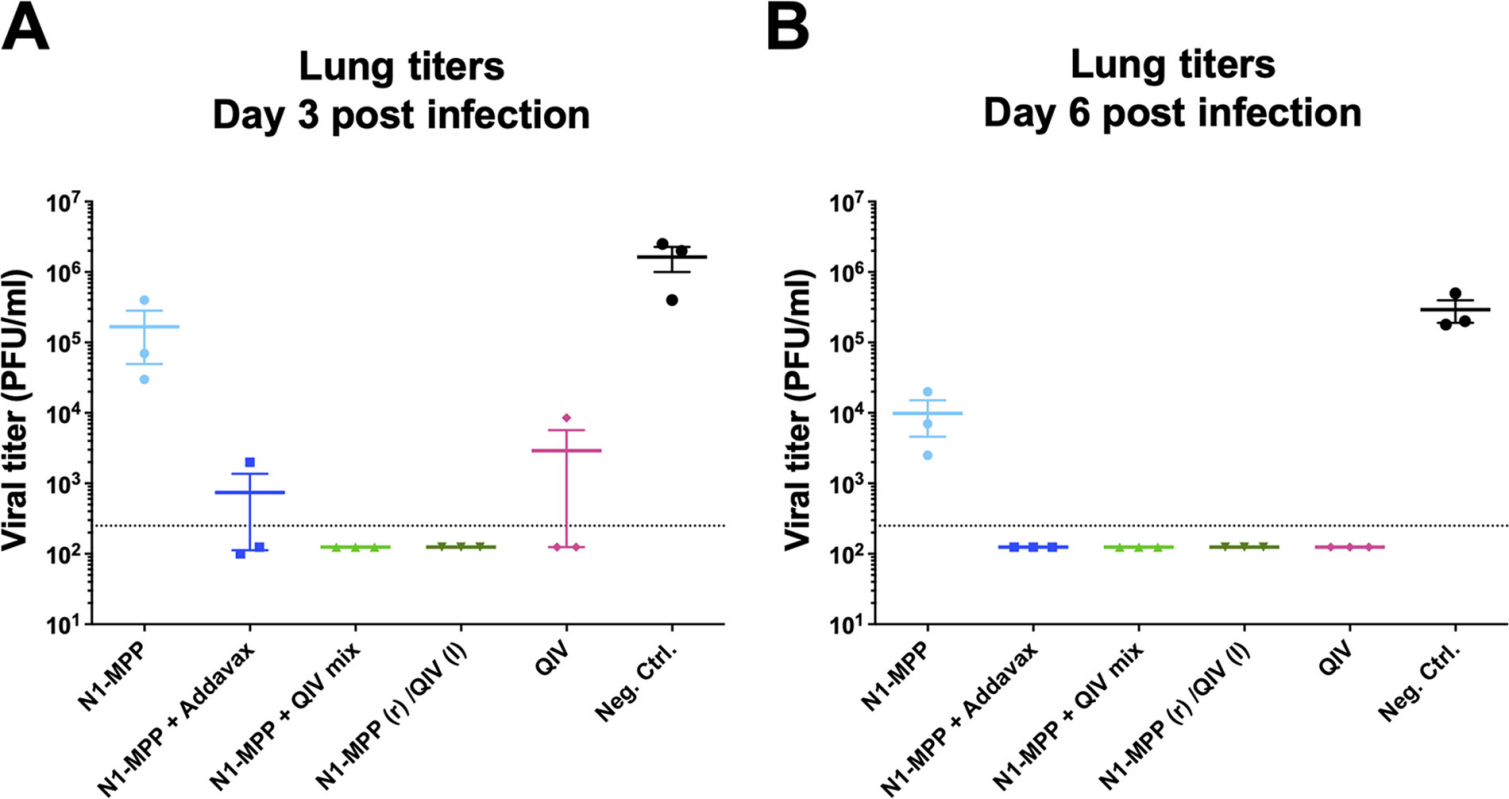
Quantification of viral loads in lung tissues of mice vaccinated with N1-MPP. Mice were vaccinated in a prime/boost regimen and then challenged with 0.5× the LD_50_ of A/Singapore/GP1908/2015 (H1N1). (A) Lungs were collected at day 3 postinfection. High viral titers were detected in the vaccinated N1-MPP and negative-control (recombinant B-HA protein) groups, and lower titers were detected in the groups N1-MPP plus AddaVax and QIV. (B) Lungs collected at day 6 postinfection. Viral loads in the groups N1-MPP plus AddaVax and QIV cleared out, and the titers in the N1-MPP and negative-control groups remained high.

Since the NA is a crucial protein in the viral replication cycle, it was of interest to determine if antibodies could potentially inhibit the spread of virus in lung tissue. To test this, immunized mice (6 per group) were challenged with 0.5× the LD_50_ of A/Singapore/GP1908/2015 (H1N1). At day 3, a high viral load of 1.66 × 10^5^ PFU/ml was detected in the N1-MPP group, which was only an order of magnitude lower than the value for the negative-control group, with 1.63 × 10^6^ PFU/ml (Fig. 6A). In contrast, the N1-MPP AddaVax group showed a lower viral titer of 2.20 × 10^3^ PFU/ml (2 out of 3 animals showed no viral load). This was similar to the titer of the group which received the matched QIV only. Groups administered the N1-MPP plus QIV mix and N1-MPP (r)/QIV (l) showed no viral load. At day 6, infectious virus remained present in the N1-MPP group (9.83 × 10^3^ PFU/ml) but was lower than in the negative-control group (2.39 × 10^5^ PFU/ml). No virus was now detected in mice vaccinated with N1-MPP plus AddaVax at this time point (Fig. 6B).

### Vaccination with N1-MPP protects even after a high-dose challenge.

**FIG 7:**
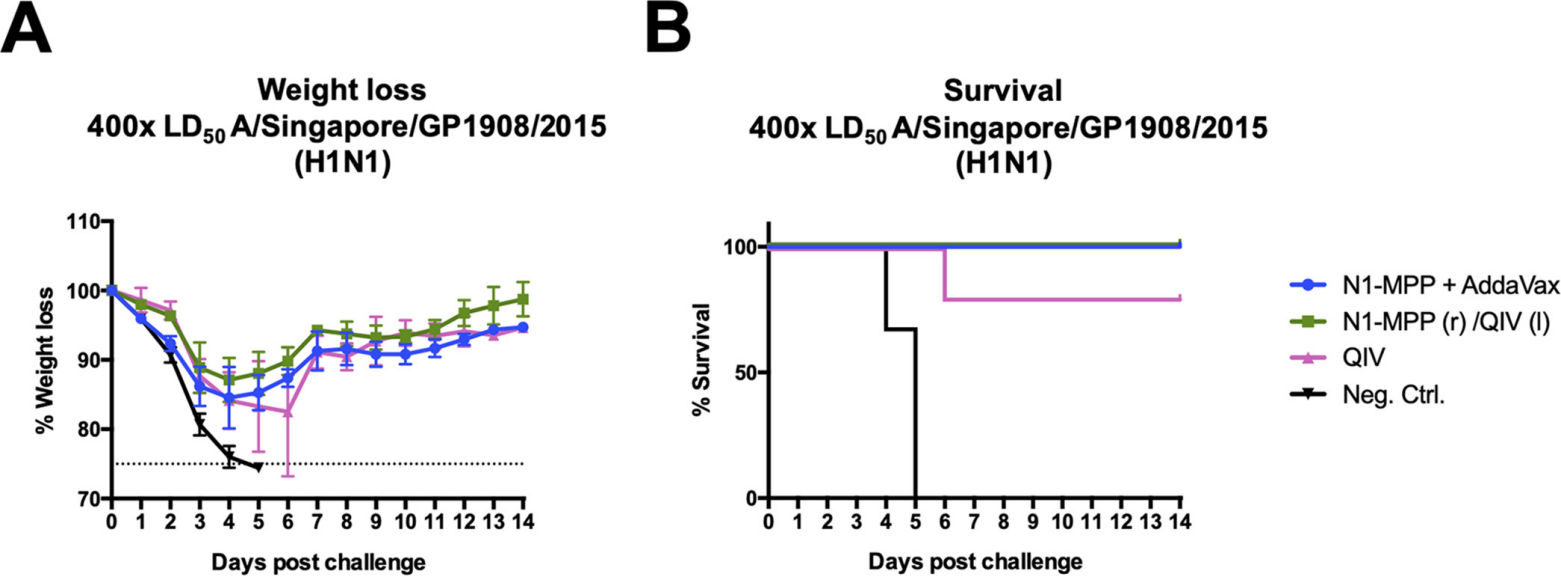
Challenge of mice in a prime/boost regimen with an elevated LD_50_ dose. (A) Weight loss curve after challenge with 400× the LD_50_ of A/Singapore/GP1908/2015 (H1N1). All vaccinated groups experienced around 10 to 15% morbidity. The dotted line indicates 75% of the initial weight, the endpoint for weight loss. (B) Survival curve. N1-MPP plus AddaVax and N1-MPP (r)/QIV (l) mice were fully protected against mortality. One mouse in the QIV group succumbed to infection on day 6. Negative-control mice succumbed to infection by day 5.

Since no substantial differences were observed during the first challenge experiment, the viral challenge dose was elevated to 400× the LD_50_ to assess the extent of potential protection induced by N1-MPP when given alone or at the same time with the QIV but in the other leg; results were compared to those for mice given the QIV only (Fig. 7A) (5 mice per group). However, all groups experienced a 10 to 15% weight loss, and one mouse in the QIV group succumbed to infection on day 6 (Fig. 7B).

### Admixture with different QIVs consistently results in blunted anti-NA responses.

**FIG 8:**
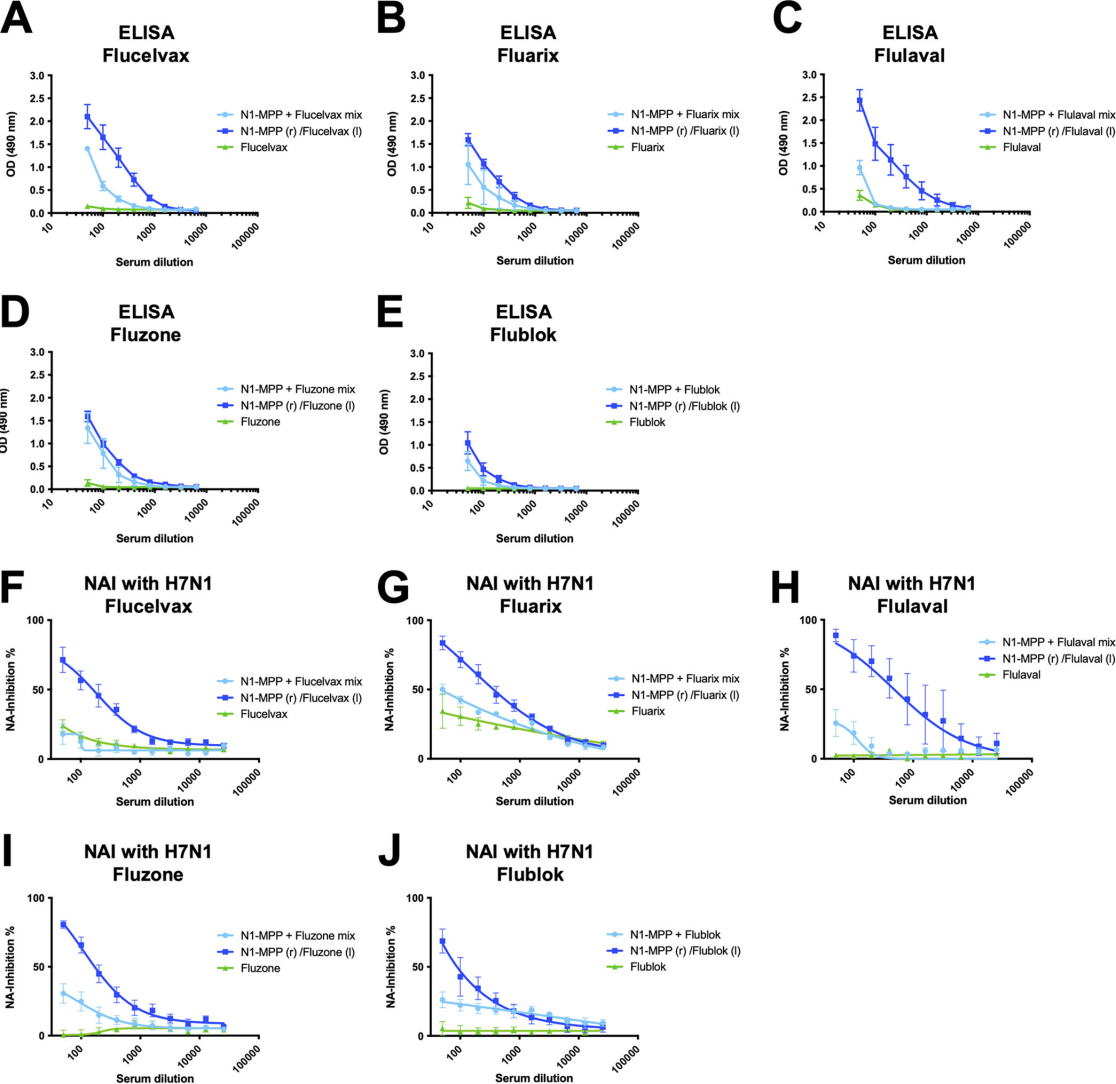
Experiment to assess characteristics of serum samples of mice vaccinated with N1-MPP and different QIVs. (A to E) ELISA against recombinant N1-VASP of mice vaccinated with Flucelvax (A), Fluarix (B), Flulaval (C), Fluzone (D), and Flublok (E). Mice were vaccinated with either N1-MPP (r)/QIV (l), N1-MPP plus QIV mix, or QIV only. Groups which received N1-MPP and QIV in different extremities appeared to have a higher antibody response to the NA than groups which received N1-MPP plus QIV mixed in one vaccine shot. (F to J) Serum NI against reassortant H7N1 virus, containing the N1 of A/Michigan/45/2015, of mice vaccinated with Flucelvax (F), Fluarix (G), Flulaval (H), Fluzone (I), and Flublok (J). Mice that received N1-MPP and QIV in different legs displayed higher NI activity.

As described above, admixing of N1-MPP and the QIV led to a strong reduction in the anti-NA antibody response compared to that after administration of N1-MPP alone or administration of N1-MPP and the QIV in different legs. To determine if this was specific to the brand of QIV used, we set out to test these combinations with other available seasonal influenza virus vaccines as well. We vaccinated mice with different QIVs (Fluarix, Flulaval, Fluzone, Flublok). Mice (5 per group) received N1-MPP plus the QIV admixed in one vaccination shot, N1-MPP (r)/QIV (l), or the QIV alone. The serum obtained after the boost was tested for reactivity to recombinant N1-VASP protein via an ELISA. We observed that all additional QIVs showed the trend that was previously seen with Flucelvax (Fig. 8). Again, when N1-MPP and the QIV were administered in different extremities of the mouse at the same time (N1-MPP [r]/QIV [l]), the antibody response appeared to be higher than in the groups where N1-MPP and the QIV were mixed together and given in one injection (Fig. 8A to E). The same trend was apparent when we analyzed the NI activities of the individual QIV groups. The groups vaccinated with N1-MPP (r)/QIV (l) induced higher NI titers than the N1-MPP plus QIV admix groups (Fig. 8F to J).

## DISCUSSION

Influenza virus vaccines protect against closely matched circulating strains, but due to antigenic drift, egg adaptations, and antigenic diversity (e.g., H3N2 clades), the vaccine often shows low effectiveness (https://www.cdc.gov/flu/vaccines-work/effectiveness-studies.htm#figure). Typically, seasonal influenza virus vaccines induce strong immune responses to the immunodominant head domain of the HA but not against the viral NA (18, 36). However, it has been shown that NA-targeted antibody responses can be highly protective and are an independent correlate of protection (28–30, 47–49). Additionally, NA and HA antigenic drift are discordant, and NA typically exhibits lower drift rates (4–6). Data from studies using animal models and MAbs isolated from humans and mice further suggest the presence of conserved epitopes in the NA (18, 44, 45, 50, 51). Therefore, enhanced immune responses against NA may act as a safety net when HAs of the vaccine and circulating strains are mismatched. Furthermore, increased anti-NA immunity may also provide some protection from emerging pandemic strains, especially if they carry an N1 or N2 neuraminidase subtype (like H2N2, H5N1, H5N2, H6N1, H7N1, H7N2, or H9N2). The NA content in seasonal vaccines is not standardized, the NA is likely presented with suboptimal structural integrity, and the HA in seasonal vaccines may exert immune dominance over the NA (32–34). A potential solution is to express stable, recombinant, tetrameric NA and either supplement it with the vaccine by admixture or give it as a standalone vaccine (likely in addition to seasonal influenza virus vaccines). While full-length NA can be expressed recombinantly and purified from cells via detergent extraction, this method suffers from low yields (39). Expression of recombinant, soluble NA head domains leads to much higher yields but requires the addition of an N-terminal tetramerization domain (43). Here, we designed three constructs featuring tetramerization domains from measles virus phosphoprotein, Sendai virus phosphoprotein, and an Arabidopsis thaliana transcription factor. We found that the N1-MPP construct, which contains the measles virus phosphoprotein tetramerization domain, showed the best characteristics during initial testing. N1 NA expressed with this tetramerization domain was intact and resembled structures of N1 NA that were previously solved.

This construct was then tested in different formulations in the mouse model. These formulations included adjuvanted and nonadjuvanted N1-MPP, but we also admixed it with regular QIV since this would be one real-world use case. In addition, since we worried about antigenic competition with HA, we also tested injection of the QIV in one leg of the mouse and N1-MPP in the other leg for comparison to the admixture. N1-MPP on its own induced full protection against lethal viral challenge, and this protection was enhanced when adjuvant was used. In fact, when we considered inhibition of virus replication in the lung, the adjuvanted N1-MPP was on par with the matched QIV. When NA was given on its own, we also found a good correlation between NI titers and protection. When both the QIV and recombinant NA were given, the protection afforded by the matched HA was likely strong enough to mask any additional effects by NA-based immunity. Furthermore, we found that admixing N1-MPP with QIV diminished the response to NA but had no effect on the response to H1 HA. To ascertain that this was not an artifact of one specific QIV formulation, we tested the impact of admixing with different brands of seasonal influenza virus vaccines and found the same impact on NA immunity. While this effect may indeed be due to immune dominance of HA over NA, it may also be caused by biophysical interactions and, potentially, degradation or structural disintegration of recombinant NA in these vaccine formulations. In fact, historic studies report that admixing NA with inactivated monovalent vaccines significantly enhanced responses to NA (35), and it is currently unclear why our results differ from historic results. One possibility is that historic vaccines contained more innate immune receptor agonists than the current QIV. The innate immune receptor agonist can act as an adjuvant, and it is possible that further adjuvantation of the QIV and recombinant NA combinations may break the immunodominance of HA and enhance the anti-NA response. This information is important and can guide clinical trial design for N1-MPP, which will be further developed as part of NIAID’s CIVIC program. Of note, we will further refine this vaccine candidate. Both the thrombin cleavage site and the hexahistidine tag will be removed for the final GMP-manufactured N1-MPP product to be tested in clinical trials. In addition, while AddaVax is conveniently available for preclinical experiments and has demonstrated an adjuvant effect, other adjuvants more suited for use in humans will need to be selected. A suitable formulation for long-term storage will also need to be worked out. Frozen or lyophilized formulations may suffice for phase I and II trials, but a liquid formulation would be desirable for phase III trials and a final product. Finally, low reactivity with measles-immune sera supports the use of this construct as a novel vaccine candidate. This study demonstrates that N1-MPP is able to elicit strong and protective anti-N1 immunity. The strategy used here to generate the antigens is also amenable for the production of recombinant N2 and influenza B virus neuraminidase, and ideally, a future NA-based vaccine will contain N1, N2, and influenza B virus NA to provide optimal protection against seasonal influenza. While it is currently unclear what a final product would look like, admixture with the QIV, administration of the NA vaccine separately (in a different arm or at a different time), and admixture of recombinant NA with a recombinant HA vaccine are possible development avenues.

## MATERIALS AND METHODS

### Cells.

BTI-TN-5B1-4 (*Trichoplusia ni*, High Five) cells were passaged in Express Five medium (Gibco) supplemented with 1% l-glutamine (Gibco) and 1% penicillin/streptomycin antibiotic mix (100 U/ml of penicillin, 100 μg/mL streptomycin; Gibco). Sf9 (Spodoptera frugiperda) cells were passaged in *Trichoplusia ni* medium-Fred Hink (TNM-FH; Gemini Bioproducts) supplemented with 10% fetal bovine serum (FBS; Gibco), 1% Pluronic-F68 (Sigma-Aldrich), and 1% penicillin/streptomycin antibiotic mix. To passage the baculoviruses in Sf9 cells, the medium was switched to TNM-FH medium containing 3% FBS, 1% Pluronic F-68, and 1% penicillin/streptomycin antibiotics. Madin-Darby canine kidney (MDCK) cells (ATCC CCL-34) were passaged in Dulbecco’s modified Eagle’s medium (DMEM; Gibco) supplemented with 10% FBS, 1% penicillin/streptomycin antibiotic mix, and 1% 4-(2-hydroxyethyl)-1-piperazineethanesulfonic acid (HEPES, Gibco). The challenge virus, A/Singapore/GP1908/2015 (H1N1), was grown in 10-day-old embryonated chicken eggs (Charles River Laboratories), and titers were determined using a standard plaque assay (24). The virus is a reassortant virus containing the glycoproteins of A/Singapore/GP1908/15 (pH1N1, IVR-180) and the internal proteins of A/Texas/1/77 (H3N2). The virus was sourced from the National Institute for Biological Standards and Control.

### Construct design and protein expression.

The recombinant N1-MPP (measles virus phosphoprotein tetramerization domain, 75 amino acids [aa] [52]), N1-SPP (Sendai virus phosphoprotein, 114 aa [53]), N1-SEP (leucine zipper from SEPPALLATA-like MADS domain transcription factor from Arabidopsis thaliana, 85 aa [54]), and N1-VASP (vasodilator-stimulated phosphoprotein [43]) constructs, containing the N1 protein from A/Michigan/45/2015 (globular head domain, aa 82 to 471, N1 numbering), were expressed using the baculovirus expression system (55). The constructs were designed to have an N-terminal signal peptide, followed by a hexahistidine purification tag, the respective tetramerization domain, a thrombin cleavage site, and the N1 globular head domain, which contains the enzymatic active site. For some animal experiments, a recombinant H1 protein of A/Michigan/45/2015 H1N1 and a B-HA protein of B/Malaysia/2506/2004 were also used. The construct was designed as described previously (56). The baculoviruses were passaged in Sf9 cells to obtain higher titers and then used to infect High Five cells for protein expression (55). Recombinant proteins were purified 72 h postinfection from the High Five cell culture supernatant as described previously (55). The protein concentrations were measured using Quick Start Bradford 1× dye reagent (Bio-Rad), and proteins were stored at −80°C until further usage.

### SDS-PAGE.

To determine protein purity and integrity, the N1 proteins were loaded on SDS-PAGE gels (4 to 20% Mini-PROTEAN TGX precast protein gel; Bio-Rad). Here, 1.5 μg of protein was mixed 1:1 with 2× Laemmli loading buffer (Bio-Rad) under reducing or nonreducing conditions and heated at 95°C for 15 min. For reducing conditions, the loading buffer was supplemented with 5% β-mercaptoethanol. For nonreducing conditions, the samples were run in the absence of reducing agents. To visualize protein tetramerization, samples were treated with the cross-linker bis-sulfosuccinimidyl suberate (BS3; ThermoFisher) according to the manufacturer’s instructions. A/Michigan/45/2015 N1-VASP (Mich15-VASP) was included under all three conditions as a positive control. The proteins were visualized by staining with Coomassie blue (ThermoFisher) for 1 h at room temperature (RT).

### NA Star assay.

To measure the enzymatic activities of the respective proteins, an NA-Star assay was performed using the NA-Star influenza neuraminidase inhibitor resistance detection kit (ThermoFisher) as per the manufacturer’s instructions. In this assay, the proteins were diluted to a starting concentration of 10 μg/mL and then serially diluted 1:3 across the plate. The Mich15 N1-VASP protein was used as a positive control. The signal was based on the luminescence readout and measured using a Synergy H1 hybrid multimode microplate reader (BioTek). The data were analyzed using GraphPad Prism 7.

### Animal work.

Animal experiments were performed under protocols approved by the Mount Sinai Institutional Animal Care and Use Committee at the Icahn School of Medicine at Mount Sinai. Female 6- to 8-week-old BALB/c mice (5 per group) were vaccinated with either (i) 3 μg of N1-MPP, (ii) 3 μg of N1-MPP mixed 1:1 with the adjuvant AddaVax (InvivoGen), (iii) the seasonal QIV (1 μg of each HA) Flucelvax (Seqirus) mixed with 3 μg of N1-MPP, (iv) the seasonal QIV (1 μg per HA) in the left leg and 3 μg of N1-MPP in the right leg administered at the same time (N1-MPP [r]/QIV [l]), (v) the seasonal QIV (1 μg per each HA), or (vi) 3 μg of irrelevant HA protein (B/Malaysia/2506/24). After 21 days, the vaccination was repeated, and after a subsequent 21 days, mice were intranasally challenged with 25× the 50% mouse lethal dose (mLD_50_) of A/Singapore/GP1908/2015 (IVR-180) H1N1 virus diluted in phosphate-buffered saline (PBS). Weight loss was monitored over a 14-day time course, and any mouse which lost more than 25% of its initial body weight was euthanized. Blood was obtained from each mouse by submandibular bleeding on day 21 and day 42 after priming for serological analysis. To determine differences in viral loads, mice were vaccinated in a prime-boost regimen as described above. Twenty-one days after the boost, mice were intranasally challenged with 0.5× the mLD_50_ of A/Singapore/GP1908/2015 H1N1. Lungs were collected at day 3 and day 6 postinfection and homogenized, and the viral load was determined via plaque assay (24). To determine if N1-MPP vaccination protects even at higher challenge doses, mice (5 per group) were vaccinated with a prime/boost regimen in the following groups: 3 μg N1-MPP plus AddaVax, 3 μg N1-MPP (right leg) plus the QIV (left leg, 1 μg per HA), the QIV (1 μg per HA), and 3 μg irrelevant HA protein. The mice were challenged with 400× the mLD_50_ of A/Singapore/GP1908/2015 H1N1. In an additional experiment, mice (5 per group) were vaccinated with different QIVs: Fluarix (GlaxoSmithKline; lot NY4EK), Flulaval (GlaxoSmithKline; lot 4TM23), Fluzone (Sanofi Pasteur; lot UT7006MA), or Flublok (Protein Sciences; lot QFAA2006). For this experiment, mice were bled for serological analysis on day 21 and day 42 after being primed.

### Human serum samples.

Residual deidentified serum samples were obtained during a phase II safety and immunogenicity study of typhoid conjugate vaccine among children in Ouagadougou, Burkina Faso (IRB no. HP-00081030 [57]). Children 9 through 11 months of age enrolled in this clinical trial were administered measles-rubella vaccine as per the Burkina Faso Expanded Program on Immunization schedule. Serum samples from day 0 (prevaccination) and day 28 (postvaccination) from participants whose parent/guardian consented to future use of specimens on the informed consent were tested for N1-MPP reactivity by ELISA, as described below.

### ELISA.

Highly binding polystyrene 96-well plates (Immulon 4 HBX plates; ThermoFisher) were coated with 50 μL/well of the respective recombinant protein at a concentration of 2 μg/mL in PBS (pH 7.4; Gibco) overnight at 4°C. The following day, the coating solution was removed and the plates were blocked with 100 μL/well of 3% milk (AmericanBio) in PBS with 0.1% Tween (PBST) for 1 h at RT. The blocking solution was removed, and the monoclonal antibodies were diluted in 1% milk/PBST to a starting concentration of 30 μg/mL; then they were serially diluted 1:3 across the plate and incubated for 1 h at RT. To assess correct folding of the proteins as well as representation of major antigenic epitopes, human MAbs 1G01 (broad anti-NA [44]) and 1000-1D05 (anti-N1 [45]) as well as mouse MAb 4A5 (anti-N1 [18]) were used. As a negative control, an anti-Lassa virus glycoprotein MAb (KL-AV-1A12 [58]) was included. For ELISAs utilizing serum as a primary antibody, the samples were diluted (1:50) in 1% milk/PBST, serially diluted 1:3, and then incubated for 2 h at RT. After primary antibody incubation, the plates were washed 3× with PBST and incubated with the respective secondary antibody, anti-mouse IgG H&L peroxidase-conjugated (Rockland) or anti-human IgG Fab-specific horseradish-peroxidase (HRP) (Sigma-Aldrich). The secondary antibodies were diluted 1:3,000 in 1% milk/PBST and added to the plate (100 μL/well) for 1 h at RT. The plates were washed three times with PBST, and 100 μL/well of SigmaFast *o*-phenylenediamine dihydrochloride (OPD) developing solution (Sigma-Aldrich) was added for 10 min. The reaction was stopped by adding 50 μL/well of 3 M hydrochloric acid (HCl). The plate was read using a Synergy H1 hybrid multimode microplate reader (BioTek) at an optical density of 490 nm. The data were analyzed using GraphPad Prism 7. For calculation of area under the curve (AUC) values, a cutoff value of the average of the optical density (OD) values of blank wells plus 3 standard deviations was established for each plate.

### NI assay.

To test if vaccination with N1-MPP induces serum antibodies with NI activity, an NI assay was performed as described previously (59). Flat-bottom nonsterile 96-well plates (Immulon 4 HBX plates; ThermoFisher) were coated with 150 μL/well of 50-μg/mL fetuin (Sigma-Aldrich) at 4°C overnight. The following day, serum samples were heat inactivated for 1 h at 56°C and then diluted 1:100 in PBS and serially diluted 1:3 on a separate 96-well plate. A reassortant virus containing an exotic H7 HA (A/Shanghai/2/2013) and the N1 of A/Michigan/45/2015 was diluted in PBS and added to the serum dilution at 2× the 50% effective concentration (EC_50_). The serum-virus mixture was incubated for 1 h 45 min, with shaking at RT. At the same time, the fetuin-coated plates were washed three times with PBST and then blocked with 200 μL/well of 5% bovine serum albumin (BSA)/PBS for 1 h at RT. The plates were washed three times with PBST, and 100 μL of the serum-virus mixture was transferred and incubated at 37°C for 2 h. The plates were washed three times with PBST and then incubated with 100 μL/well of peanut agglutinin conjugated to HRP (PNA; Sigma-Aldrich) at a concentration of 5 μg/mL for 1 h 45 min at RT. The plates were washed three times with PBST, and 100 μL/well of SigmaFast OPD developing solution was added. After 7 min of incubation, the reaction was stopped by adding 50 μL/well of 3 M HCl. The OD was measured at 490 nm using a Synergy 4 plate reader (BioTek). The data were analyzed using GraphPad Prism 7 software, and values are expressed as percent inhibition.

### Plaque assay.

For virus titration and for determination of viral load in murine lung tissues (collected on days 3 and 6 postchallenge with 0.5× the LD_50_ of A/Singapore/GP1908/2015 H1N1), standard plaque assays were performed. Briefly, confluent monolayers of MDCK cells were infected with different sample dilutions of virus/homogenized lung tissue ranging from 1:10 to 1:1,000,000 and were diluted in 1× minimal essential medium (1% penicillin/streptomycin antibiotic mix, 1% HEPES, 1% l-glutamine, and 1% sodium-bicarbonate [Gibco]) for 1 h at 37°C. Afterwards, an overlay containing 2% Oxoid agar (ThermoFisher), H_2_O, 2× minimal essential medium, diethylaminoethyl (DEAE), and *N-p*-tosyl-l-phenylalanine chloromethyl ketone (TPCK)-treated trypsin was added to the cells. The plates were incubated at 37°C for 2 days and then fixed with 10% paraformaldehyde overnight at 4°C. Plaques were visualized by immunostaining. For this, the agar overlay was removed, and the plates were blocked with 3% milk/PBS. The blocking solution was removed, and primary antibody (anti-N1 4A5) diluted 1:3,000 in 1% milk/PBS was added for 1 h. The plates were washed three times with PBS, and secondary antibody (anti-mouse IgG H&L peroxidase-conjugated antibody [Rockland]) diluted 1:3,000 in 1% milk/PBS was added for 1 h. The plates were washed three times with PBS and developed by using KPL TrueBlue peroxidase substrate (SeraCare). The plaques were counted and analyzed using GraphPad Prism 7.

### Crystal structure determination of the recombinant N1 NA.

The recombinant N1-MPP proteins were purified 72 h postinfection from the Sf9 cell culture supernatant by metal affinity chromatography using Ni-nitrilotriacetic (NTA) resin (Qiagen) as described previously (43). For crystal structure determination, the MPP tetramerization domain and the His_6_ tag were removed from the N1-MPP with thrombin and purified further by size exclusion chromatography on a HiLoad 16/90 Superdex 200 column (GE Healthcare) in 20 mM Tris, pH 8.0, 150 mM NaCl, and 0.02% NaN_3_. The purified NAs were quantified by optical absorbance at 280 nm, and their purity and integrity were analyzed by reducing and nonreducing SDS-PAGE.

Crystallization experiments were set up using the sitting drop vapor diffusion method using automated robotic crystal screening on a CrystalMation system (Rigaku). Diffraction-quality crystals for the N1 NA were obtained by mixing 0.1 μL of the NA protein at 7.2 mg/mL in 20 mM Tris, pH 8.0, 150 mM NaCl, and 0.02% (vol/vol) NaN_3_ with 0.1 μL of the well solution in 0.2 M calcium acetate and 20% (wt/vol) polyethylene glycol 3350 at 20°C. The N1 NA crystals were flashed-cooled at 100 K with 10% (wt/vol) ethylene glycol added as a cryo-protectant. Diffraction data were collected at synchrotron beamlines (see Table S1 in the supplemental material). Data for all crystals were integrated and scaled with HKL2000 (60). Data collection statistics are summarized in Table S1. The crystal structure was determined by molecular replacement (MR) using the program Phaser (61). The N1 NA structure was determined using the N1 head domain structure from A/California/04/2009 (H1N1) (Protein Data Bank [PDB] accession no. 3NSS). Initial rigid-body refinement was performed in REFMAC5 (62), and subsequently, rigid-body and group ADP refinement were carried out in Phenix (63). Between rounds of refinements, model building was examined and modified with the program Coot (64). Final statistics are summarized in Table S1. The quality of the structure was analyzed using the JCSG validation suite (www.jcsg.org) and MolProbity (65). All figures were generated with PyMOL (www.pymol.org).

### Statistical analysis.

Statistical analysis was performed in GraphPad Prism 9.0.1. NI titers were analyzed using one-way analysis of variance (ANOVA) and corrected for multiple comparisons.

### Data availability.

The atomic coordinates and structure factors for the N1 head domain from A/Michigan/45/2015 (H1N1) have been deposited in PDB under accession no. 7S0I.
