# Supplementary material for: A Novel Recombinant Influenza Virus Neuraminidase Vaccine Candidate Stabilized by a Measles Virus Phosphoprotein Tetramerization Domain Provides Robust Protection from Virus Challenge in the Mouse Model
Source: mBio. 2021 Nov 23;12(6):e02241-21. doi: 10.1128/mBio.02241-21 (PMC8609353; doi:10.1128/mBio.02241-21)
Supplement: TABLE S1 [file mbio.02241-21-st001.docx]

**Table S1.** **Data collection and refinement statistics**

| Data set | **N1 head from N1-MPP** | |
| --- | --- | --- |
| **Data Collection** |  | |
| X-ray source | APS 23-IDD |  |
| Space group | P42_1_2 | |
| Unit cell (Å) | *a* = *b* = 92.1,  *c* = 104.9 | |
| Resolution (Å)^a^ | 46.1-2.90 (2.95-2.90) | |
| Unique reflections | 9,713 | |
| Redundancy^a^ | 6.8 (5.8) | |
| Average I/σ(I)^a^ | 4.0 (1.0) | |
| Completeness^a^ | 91.5 (93.4) | |
| *R*_sym_^a,b^ | 0.35 (0.87) | |
| *R*_pim_^a,b^ | 0.14 (0.36) | |
| CC_1/2_^a^ | 0.953 (0.810) | |
| No. molecules per ASU^c^ | 1 | |
|  |  | |
| **Refinement** |  | |
| Reflections in refinement | 9,660 | |
| Refined residues | 387 | |
| Refined waters | 4 | |
| *R*_cryst_^d^ | 0.292 | |
| *R*_free_^e^ | 0.321 | |
| *B*-values (Å^2^)  Protein  Water | 35  37 | |
| Wilson *B*-values (Å^2^) | 59 | |
| Ramachandran values (%)^f^ | 96.1, 0.5 | |
| r.m.s.d. bond (Å) | 0.009 | |
| r.m.s.d. angle (deg.) | 1.48 | |
| PDB code | AAAA | |

^a^ Parentheses denote outer-shell statistics.

^b^ *R*_sym_ = ∑*_hkl_*∑*_i_* |*I_hkl,i_* - <*I_hkl_*>| /∑*_hkl_*∑*_i_* *I_hkl,i_* and *R*_pim_ = ∑*_hkl_*[1/(*N*-1)]^1/2^∑*_i_* |*I_hkl,i_* - <*I_hkl_*>| /∑*_hkl_*∑*_i_* *I_hkl,i_*, where *I_hkl,i_* is the scaled intensity of the i^th^ measurement of reflection *h*, *k*, *l*, < *I_hkl_*> is the average intensity for that reflection, and *N* is the redundancy. R_pim_ = Σ*_hkl_* (1/(n-1))^1/2^ Σ*_i_* | *I_hkl,i_* - *<I_hkl_>* | / Σ*_hkl_* Σ*_i_ I_hkl,i_*, where n is the redundancy

^c^ No. molecules refers to the number of NA protomers per asymmetric unit (ASU).

^d^ *R*_cryst_ = ∑*_hkl_* |*F_o_* - *F_c_*| / ∑*_hkl_* |*F_o_*|, where *F_o_* and *F_c_* are the observed and calculated structure factors.

^e^ *R*_free_ was calculated as for *R*_cryst_, but on 5% of data excluded before refinement.

^f^ The values are percentage of residues in the favored and outliers regions analyzed by MolProbity (67).
