# Supplementary figures and images for: A Novel Recombinant Influenza Virus Neuraminidase Vaccine Candidate Stabilized by a Measles Virus Phosphoprotein Tetramerization Domain Provides Robust Protection from Virus Challenge in the Mouse Model
Source: mBio. 2021 Nov 23;12(6):e02241-21. doi: 10.1128/mBio.02241-21 (PMC8609353; doi:10.1128/mBio.02241-21)

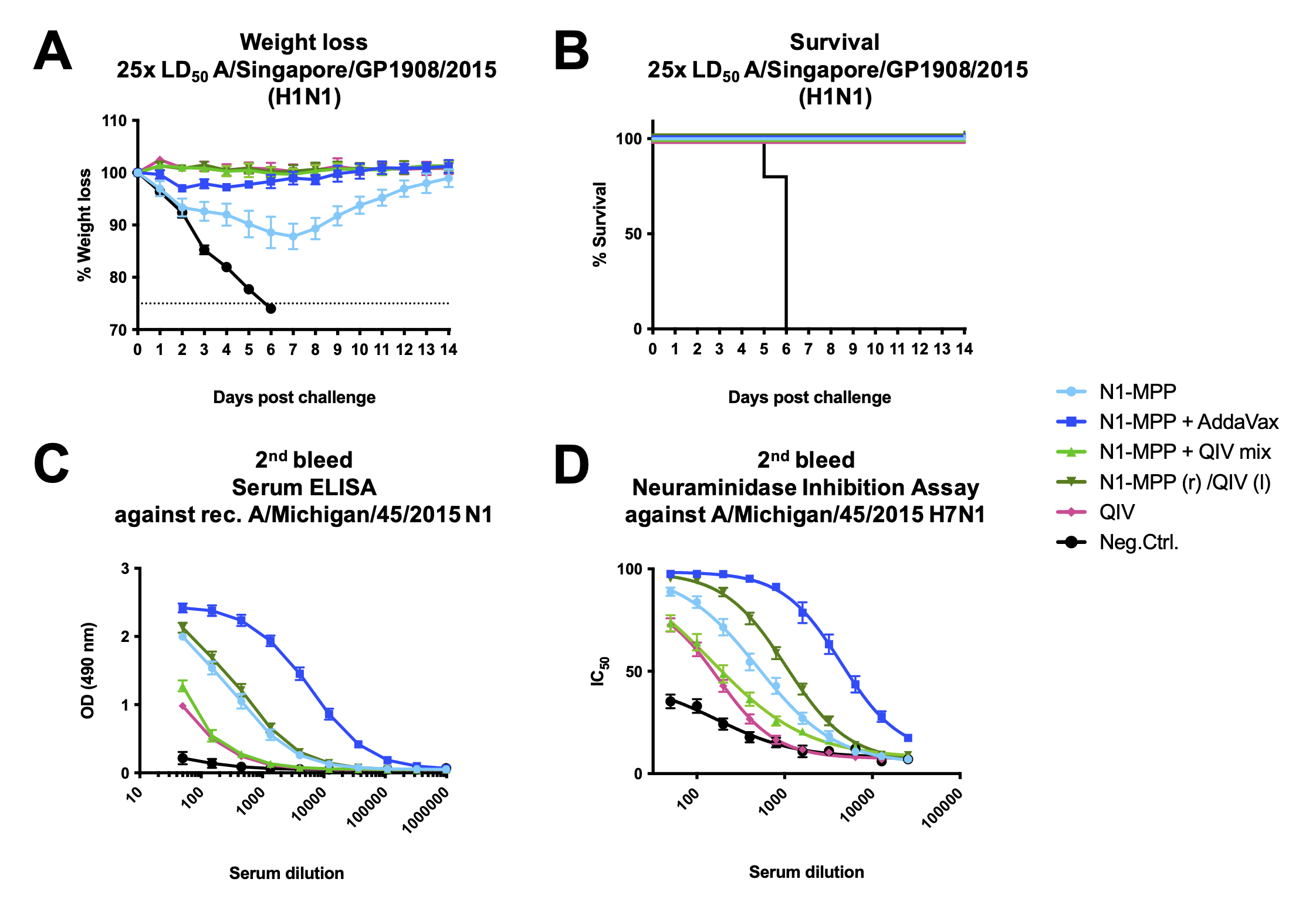

Supplement: FIG S1 [file mbio.02241-21-sf001.tif]

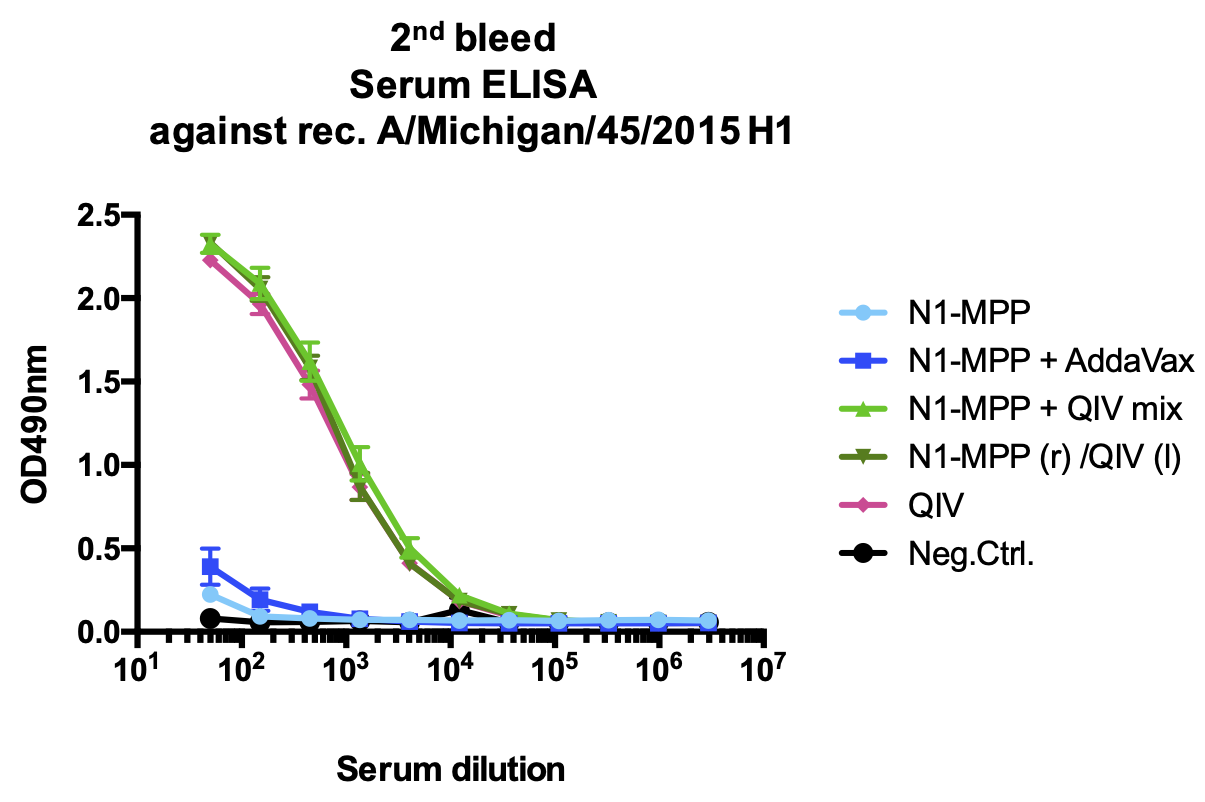

Supplement: FIG S2 [file mbio.02241-21-sf002.tif]
